# Interaction of social support and depressive symptoms on antiretroviral therapy adherence among people living with HIV in South Africa

**DOI:** 10.4102/hsag.v29i0.2271

**Published:** 2024-06-06

**Authors:** Muziwandile Q. Luthuli, Johannes John-Langba

**Affiliations:** 1 Discipline of Social Work, School of Applied Human Sciences, University of KwaZulu-Natal, Durban, South Africa

**Keywords:** ART adherence, depressive symptoms, HIV, social support, South Africa

## Abstract

**Background:**

Depression consistently emerges as a significant predictor of poor antiretroviral therapy (ART) adherence among adult people living with human immunodeficiency virus (PLHIV). However, a gap exists regarding how social support and depressive symptoms can interact to influence ART adherence among adult PLHIV in South Africa (SA).

**Aim:**

To investigate the interaction between social support and depressive symptoms on ART adherence among adult PLHIV.

**Setting:**

A tertiary hospital in Durban, KwaZulu-Natal province of SA.

**Methods:**

Utilising a quantitative cross-sectional research design along with time location sampling technique (TLS); the study recruited 201 adult patients enrolled in an ART programme.

**Results:**

The results indicated that depressive symptoms were significantly associated with ART adherence with and without the interaction (B = -0.105; odds ratios [OR] 0.901; 95% confidence intervals [CI] = 0.827, 0.981; *p* = 0.016), while social support was not significantly associated with ART adherence (B = 0.007; OR 1.007; 95%CI = 0.989, 1.025; *p* = 0.475). However, a statistically significant interaction was found between social support and depressive symptoms (B = -0.006; OR 0.994; 95%CI = 0.989, 1.000; *p* = 0.037) on ART adherence.

**Conclusion:**

Based on the results, depressive symptoms significantly influenced ART adherence. However, social support did not buffer the adverse effects of clinical depression associated with poor ART adherence.

**Contribution:**

This study provides an evidence-based approach to address gaps in the mental health and social well-being of PLHIV in the context of ART adherence.

## Introduction

The global spread of the human immunodeficiency virus (HIV) has led to considerable human suffering, as people living with the disease are more susceptible to different detrimental health conditions. The number of people living with human immunodeficiency virus (PLHIV) globally has also been increasing, and this indicates that HIV remains a serious global health concern. For instance, at the global level, the number of PLHIV increased from 34 million in 2010 to 38.4 million in 2021, with almost three-quarters (74.7%) of the people estimated to access antiretroviral therapy (ART) (Joint United Nations Programme on HIV/AIDS [UNAIDS] 2022). Moreover, acquired immunodeficiency syndrome (AIDS)-related deaths have decreased by 52%, from 1.4 million people in 2010 to an estimated 650 000 in 2021 (UNAIDS 2022). On the other hand, the increase in ART access has also been a vital factor in reducing the number of new HIV infections by 32%, from 2.2 million in 2010 to 1.5 million in 2021 (UNAIDS 2022). Despite this significant progress in prevention and treatment, the global HIV epidemic continues to cause a disproportionate burden of disease among vulnerable groups. This is particularly prevalent among those living in low-and middle-income countries (Shao & Williamson 2011).

Moreover, the sub-Saharan Africa (SSA) region stands out for its tremendously higher HIV prevalence compared to other global regions, with South Africa (SA) demonstrating this trend. For example, SA experienced an increase in the population living with HIV from 7.8 million people in 2020 to 8.5 million people in 2021, maintaining its position as the epicentre of the HIV/AIDS pandemic (Statistics South Africa [Stats SA] 2022; UNAIDS 2020). The prevalence rate of HIV among adults aged 15–49 years in SA surged from 18.3% in 2021 to 19.6% by July 2022 (Stats SA 2022). This surpassed the overall HIV prevalence rate of 13.9% estimated for the South African population in the mid-2022 by Stats SA (2022). In the same vein, the distribution of HIV cases in 2021 displayed a gender imbalance, where out of the 7.3 million adult PLHIV aged 15–49 years in SA, approximately 4.8 million were women, whereas men living with HIV were 2.4 million (UNAIDS 2021). Notably, the burden of HIV was higher in women, accounting for around 24.5%, compared to men at 12.1%, indicating a substantial difference of 12.4% (UNAIDS 2021). This disproportionality in HIV prevalence between women and men is because of a variety of factors including but not limited to gender-based violence and unequal access to healthcare services such as HIV testing, prevention and treatment services (Muyanga et al. 2023; UNAIDS 2020).

In addition, men tend to have higher rates of risky sexual behaviours as well as lower rates of HIV testing, and treatment adherence than women, which further contributes to the gender gap in HIV prevalence in SA (Wechsberg et al. 2013). Moreover, the national coverage of ART among PLHIV increased by just 2% in 1 year, with 5.5 million people translating to 74% in 2021 (UNAIDS 2021), up from 72% in 2020 (UNAIDS 2020). This indicates the slow progress being made in providing access to ART for PLHIV, as the mere 2% increase within a year fell significantly below the required 18% rise to attain the target of 90% for universal access to ART (South African National AIDS Council 2017). As the HIV pandemic continues to ravage KwaZulu-Natal (KZN) province, its HIV prevalence rate remains the highest in the entire country of SA. For example, in 2022, HIV prevalence in KZN was estimated at 18.3% (KwaZulu-Natal Department of Health [KZN DoH 2022]), compared to the national prevalence of 19.6% (Stats SA 2022). Despite this, HIV prevalence in KZN has decreased gradually over the past few years, from 20.6 % in 2017 (HSRC 2018) to 18.3% in 2022 (KZN DoH 2022). This demonstrates that comprehensive HIV prevention and treatment options efforts in KZN have been successful and should be continued to further reduce HIV prevalence across the region. KwaZulu-Natal, for instance, had over 2 million PLHIV in 2021, with 1.7 million people reported to be on ART in the province. Despite the high numbers of those living with HIV/AIDS in KZN, the province still managed to provide sufficient ART treatments to those in need. This accounts for approximately 85% of the total PLHIV in the KZN province.

Despite wide access to ART, HIV care retention remains unsatisfactory in SA. The data from previous research suggest that while ART coverage has increased, HIV care retention has remained relatively low. This indicates that quality HIV care still poses challenges in SA. In SA, the loss to follow-up (LTFU) among PLHIV has been constantly estimated at between 20% and 30% of PLHIV (Plazy et al. 2014). For instance, a study conducted in KZN province at a rural hospital revealed a rate of 25.2% for LTFU, of which 0.9% of these patients died 12 months after ART initiation (Arnesen, Moll & Shenoi 2017). Another study conducted by Nyakato et al. (2022), across three South African provinces reported that over half of their study population (51.9%) were LTFU 1 year after ART initiation. These findings suggest that PLHIV in SA face a high risk of LTFU and that this risk can even be exacerbated in resource-limited settings. This further suggests that there are substantial gaps in existing care for PLHIV in SA, highlighting the need for improved retention strategies to ensure long-term care and treatment. Mental health disorders can interact with social, economic and cultural factors in different contexts (Alegría et al. 2019), making it difficult to address all the underlying causes of poor adherence to ART.

Thus, although physical symptoms are linked to HIV/AIDS, mental health must be considered when treating PLHIV to ensure successful adherence to ART. People living with human immunodeficiency virus with mental health conditions, such as depression, may find it difficult to remember to take their medication. This can lead to an increased risk of missing doses, which can ultimately lead to virological failure. Depression is a mental disorder that can present with various symptoms such as feeling of sadness and loss of interest, and it affects how one feels, thinks and behaves and can lead to a variety of emotional and physical problems (World Health Organisation. [WHO 2023]). For instance, several studies conducted in SSA have shown that PLHIV with depressive symptoms are less likely to adhere to their ART regimens, leading to poor health outcomes (Asrat et al. 2020; Ekat et al. 2020; Shearer et al. 2018; Spielman et al. 2021; Wagner et al. 2019). As a result, it is imperative to address depression in PLHIV to ensure they are adherent to their ART regimens and maintain better health.

Moreover, South African studies have documented a vast variation in depression prevalence rates that ranges up to over one-third (37%) of all PLHIV (Ekat et al. 2020; Kitshoff, Campbell, & Naidoo 2012; Shearer et al. 2018). Three recent studies have reported various prevalence estimates for depression among PLHIV in SA: for example, in Kagee et al.’s study (2020), the rate was 24.7% (*n* = 170), while out of 688 ART users in Kagee, Saal and Sterley’s study (2021), the rate was 24.9%, and in Truong et al.’s study (2021), the prevalence rate was 12.5% (*n* = 289). These discrepancies in prevalence estimates could be because of differences in the study populations, methodologies or data-collection techniques used. It is also possible that the prevalence of depression is changing over time, and these studies are showing that trend. In addition, depression is among the three major causes of disease burden in SA and elsewhere (Kitshoff et al. 2012). As such, strategies to identify, assess and treat depression among PLHIV in SA must be prioritised to ensure better physical and mental health for this population (Van Coppenhagen & Duvenage 2019). In addition, PLHIV require social support and access to medical resources to help mitigate the risk of developing depressive symptoms.

Having reliable sources of social support can be a key factor in managing depression for those living with HIV. Social support is defined as the provision of resources and assistance by people in one’s social network (Cohen & Mckay 1984). Cohen and Mckay (1984) further state that resources and assistance can be procured through emotional (e.g., love, care and concern) or material (e.g., money, housing and food) means. For instance, a recent meta-analysis showed that PLHIV with poor social support had a higher risk of developing depressive symptoms compared to those with high social support (Weldesenbet, Kebede & Tusa 2020). The study indicated that those with a poor social support system were over two times more likely to develop depressive symptoms (Weldesenbet et al. 2020). This highlights the importance of social support for PLHIV, and why it should be an important consideration when addressing mental health issues. Adequate social support can be a powerful source of strength, giving those living with HIV/AIDS the motivation to seek out necessary healthcare resources and develop a sense of belonging and hope (Wu et al. 2018). Two general models of social support have been adopted to explain the pivotal role of social support in depressive symptoms: the direct effect model and the stress buffer model. The direct effect model posits that social support directly affects the severity of depression symptoms (Lakey & Orehek 2011), while the stress buffer model suggests that social support serves to reduce stress and, in turn, reduce the severity of depression symptoms (Yan et al. 2019). Both models are important and effective in understanding how social support can be used to alleviate depressive symptoms.

Moreover, Beck’s cognitive theory of depression is adopted in the current study which posits that cognitive biases and negative beliefs about oneself (self-schemas) are shaped by environmental stressors (Beck 1967). These self-schemas create negative thought patterns that can lead to feelings of hopelessness and helplessness, which can then lead to depression. The theory suggests that by recognising and challenging these self-schemas, individuals can disrupt the cycle of negative thinking and reduce the effects of depression. Conversely, the social cognitive theory (SCT) posits that personal factors (depressive symptoms) and environmental factors (social support) function as interacting determinants that influence aspects of behaviour (adherence to ART) for a robust behavioural change (Bandura 1986, 2001). Therefore, understanding the complex relationships between personal and environmental factors can be a crucial part of developing successful interventions for improving adherence to ART among the HIV population.

Based on a large body of evidence, social support has been shown to be an effective means of improving adherence to ART and the overall quality of life for PLHIV (Mao et al. 2019; Seffren et al. 2018; Yan et al. 2019). Extensive evidence has been established demonstrating both a positive association between social support and adherence to ART (Damulira et al. 2019; Mireles et al. 2023; Oliveira et al. 2020) and a negative association between social support and depression (Jones et al. 2021; Mao et al. 2019; Yan et al. 2019; Zamanian et al. 2021). However, very little is known about the interactive mechanism between social support, depressive symptoms and ART adherence among adult PLHIV within the South African context. This knowledge gap is particularly concerning because, without understanding the mechanism through which social support and depressive symptoms interact to influence ART adherence, interventions designed to improve adherence may be ineffective. Thus, the purpose of the study was to examine the interactive mechanisms through which social support and depressive symptoms influence ART adherence among adult PLHIV in SA. Guided by cognitive theories of depression, the buffer model and SCT, the study hypothesised the following facts among adult PLHIV in Durban, SA:

*Hypothesis 1*: Depressive symptoms predict ART adherence.

*Hypothesis 2*: There is a statistically significant interaction between social support and depressive symptoms in predicting ART adherence.

## Methods and design

### Study design, setting and population

A cross-sectional quantitative research design was used. The researcher adopted the post-positivistic paradigm, which recognises the importance of social context in constructing knowledge and posits that knowledge is constructed by people through interaction with their environment. A non-probability time location sampling (TLS) technique was used to recruit participants from an ARV clinic at a tertiary hospital in Durban, KZN. Participants who met all the inclusion criteria were selected voluntarily. The inclusion criteria include PLHIV, age ≥ 18 years, enrolled in an ART program and residents of urban communities in Durban, SA. The data were gathered between June and October 2020 during the outbreak of COVID-19 in SA.

#### Sample size determination

The sample size was determined considering both financial efficiency and statistical reliability. The minimum sample size was determined using the following formula proposed by Lemeshow et al. (1990:1).

$$\begin{array}{l} S=\frac{Z^{2}\left(1-\frac{a}{2}\right)p(1-p)}{d^{2}}\\ =\frac{{\left(1.96\right)}^{2}0.144\times 0.856}{0.05^{2}}=189.41=189 \end{array}$$ [Eqn 1]

The criteria of 50% of missing data was used to ensure that the data were reliable and that the results would be accurate. With the required sample size of 189; 222 questionnaires were completed; of these, 21 were discarded because of incomplete data exceeding 50%. Therefore, 201 questionnaires were considered valid for analysis.

## Data collection

As part of the survey questionnaire, socio-demographic data were collected (see Table 1). Nominated experts underwent blind translation of the questionnaire and informed consent form from English to IsiZulu. Participants who volunteered to complete a questionnaire first gave their written consent to participate in the study. The questionnaire took almost 25 min to complete.

**TABLE 1:** Demographics, descriptive statistics and Pearson’s Chi-squared results (*N* = 201).

Variable	Frequency	%	*M*	SD	Adherence to ART				*p* †
					High adherence		Low adherence		
					*N*	%	*N*	%	
Age	201	100	39.3	12.1	108	53.7	93	46.3	0.054
**Sex**	-	-	1.3	0.5	-	-	-	-	0.468
Male	58	28.9	-	-	29	14.5	29	14.5	-
Female	142	70.6	-	-	79	39.5	63	31.5	-
Total	-	-	-	-	108	54.0	92	46.0	-
**Marital status**	-	-	1.4	0.8	-	-	-	-	0.126
Never married	147	73.1	-	-	86	43.4	61	30.8	-
Married	36	17.9	-	-	13	6.6	23	11.6	-
Divorced	6	3.0	-	-	2	1.0	4	2.0	-
Widowed	5	2.5	-	-	2	1.0	3	1.5	-
Separated	4	2.0	-	-	2	1.0	2	1.0	-
Total	-	-	-	-	105	53.0	93	47.0	-
**Education status**	-	-	2.9	0.9	-	-	-	-	0.320
No education	16	8.0	-	-	10	5.1	6	3.1	-
Primary	36	17.9	-	-	23	11.7	13	6.6	-
Secondary	97	48.3	-	-	46	23.5	51	26.0	-
Tertiary	47	23.4	-	-	25	12.8	22	11.2	-
Total	-	-	-	-	104	53.1	92	46.9	-
**Employment**	-	-	1.5	0.7	-	-	-	-	0.033
Unemployed	132	65.7	-	-	80	40.2	52	26.1	-
Employed	41	20.4	-	-	15	7.5	26	13.1	-
Self-employed	24	11.9	-	-	10	9.4	14	7.0	-
Retired	2	1.0	-	-	1	0.5	1	0.5	-
Total	-	-	-	-	106	53.3	93	46.7	-
**Depressive symptoms**	-	-	10.2	4.6	-	-	-	-	0.003
Mild	65	32.3	-	-	37	18.5	28	14.0	-
Moderate	35	17.4	-	-	25	12.5	10	5.0	-
Moderately severe	18	9.0	-	-	11	5.5	7	3.5	-
Severe	5	2.5	-	-	5	2.5	0	0.0	-
Total	-	-	-	-	108	54.0	92	46.0	-
**Social support**	-	-	63.6	20.2	-	-	-	-	0.094
High support	102	50.7	-	-	61	31.1	41	20.9	-
Low support	94	46.8	-	-	45	23.0	49	25.0	-
Total	-	-	-	-	106	54.1	90	45.9	-

M, mean; SD, standard deviation; ART, antiretroviral therapy.

† , represents significance given between high adherent and low adherent individuals.

### Measures

#### Adherence to antiretroviral therapy: A simplified medication adherence questionnaire

The outcome variable, ART adherence was measured utilising the six-item simplified medication adherence questionnaire (SMAQ) (Knobel et al. 2002). The SMAQ was invented by Knobel et al. (2002) on the foundational basis of the Morisky’s Medication Adherence Scale. The SMAQ is a self-report measure of ART adherence which is suitable for use among adults living with HIV (Knobel et al. 2002). The SMAQ assesses various facets of patient treatment adherence, specifically addressing forgetfulness, adverse effects, routine and quantification of omissions (Knobel et al. 2002).

The SMAQ consists of four items that are dichotomous *(yes/no)*, while one additional item is open *(less/more than 2 days)*, and further one item is Likert-type *(from never to more than 10 times)*. Participants were instructed to report if over the past 3 months (*e.g. ‘do you ever forget to take your medicine?’*) on a dichotomous response option (*0 = yes/1 = no*) and with one item (*e.g. ‘Thinking about the last week, how often have you not taken your medicine?’*) on a 5-point Likert scale from 0 (*Never*) to 5 (*More than 10 times*). All items within the SMAQ measure are phrased negatively, with a need for reverse scoring in two of them. Previous studies have established high internal consistency for SMAQ in adults with HIV/AIDS with alpha coefficients above 0.70 (Knobel et al. 2002). The cut-off median value of 5 was employed to differentiate between high and low ART adherence. This approach aligns with the established median split technique, facilitating a clear demarcation between high and low groups on the variable in question (MacCallum et al. 2002). This methodological choice of dichotomising at the median enhances precision and reliability of analysis within ART adherence measure (MacCallum et al. 2002).

Values ranging from 0 to 4 were indicative of high ART adherence, while values exceeding 5 denoted comparatively low ART adherence.

#### Depressive symptoms: Patient health questionnaire

A predictor variable, depression, was measured using a self-report patient health questionnaire (PHQ-9) which consists of nine items used to assess the presence of depressive symptoms over the previous 2 weeks (Kroenke, Spitzer & Williams 2001). The PHQ has shown robust validity and internal consistency when applied with PLHIV in sub-Saharan Africa (Monahan et al. 2008). The nine items match up with the symptoms that tend to be diagnosed in accordance with the Diagnostic and Statistics Manual (DSM) and responses to each item are on a 4-point Likert-type scale ranging from 0 (‘not at all’) to 3 (‘nearly every day’). The scores of items are summed up and scores of (5–9) indicate mild depressive symptoms, whereas a score of 10 > has been found to represent major depressive symptoms (Kroenke et al. 2001). The past study by Kroenke et al. (2001), has established a high internal consistency with Cronbach’s alpha ranges from 0.86 to 0.89 in the study that consists of two various patient populations.

#### Social support: Multidimensional scale of perceived social support

A moderator variable, social support was measured using a self-report multidimensional scale of perceived social support (MSPSS) (Zimet et al. 1988). The MSPSS is a self-report scale consisting of 12 items, and these items are categorised into three-factor groups associated with each dimension of perceived social support namely the support from family (4 items), friends (4 items) and significant others (4 items) (Zimet et al. 1988). The responses to the scale are rated on a 7-point Likert scale from 1 (very strongly disagree) to 7 (very strongly agree), and the high scores on all dimensions are indicative of greater social support. The MSPSS-12 has been found to have an excellent overall internal consistency that ranges between 0.80 and 0.95 (Duru 2007).

## Data analysis

### Statistical analysis

All analyses were conducted using SPSS version 28 and predictor variables were measured as continuous variables while the outcome variable was measured as a dichotomous variable. The missing data of participants were assumed to be missing at random and the normality of the data for all variables of interest was not confirmed using the Shapiro-Wilk test; therefore, non-parametric statistics were conducted. Descriptive statistics were reported in the form of standard deviations (SD), means (M) and frequencies (*n*). We also presented rates of ART adherence, depressive symptoms and social support.

Bivariate analyses assessed the main effects of all variables on ART adherence. Spearman’s rank order correlation coefficient was used to test the association between the study variables. Pearson’s chi-square tests were performed to analyse differences in demographic characteristics between high-adherent and low-adherent participants. Multiple logistic regression was conducted to test the regression model and interaction effect. Before the interaction term was computed, both predictor variables were standardised by subtracting the mean from all original scores. This is also referred to as centring deviation scores (Aiken Reno & West 1991). The product term was computed by multiplying predictor variables together (social support × depressive symptoms) in SPSS (Aguinis, Gottfredson & Culpepper 2013).

The hypothesised predictor variables (social support and depressive symptoms) were entered into the main effect model with multiple logistic regression to test for their independent effect on the dependent variable (ART adherence). The product term (social support × depressive symptoms) was added to the second logistic regression model to test if a statistical interaction exists in predicting the outcome variable (ART adherence). To compare the proportion of unique effects contributed by each explanatory variable and the interaction term, Wald chi-squared was used. Confidence intervals (95% CI), odds ratios (OR), standardised beta coefficients (B), standard errors (SE) and *p*-values were used to examine statistically significant effects in the multiple logistic regression models. For all analyses, statistical significance was observed with a cutoff probability value of 0.05 or less.

### Validity and reliability

The results of the pilot study were used to validate the accuracy of the scales. The pilot study allowed researchers to test the scales with a sample of participants and measure the accuracy of their responses. It helped to determine if the translated data-collection tool was accurate and reliable in the population under study, and necessary changes were made to the measure before it was used in the larger study. The reliability of the scales was further validated by assessing Cronbach’s alpha coefficients score, which must meet a minimum threshold of 0.70 for the measure to be considered valid (Taber 2017). The internal consistency of MSPSS-12 was excellent (α = 0.888) and slightly higher than the score of the pilot study (α = 0.793), while that of PHQ-9 was acceptable (α = 0.768) and significantly higher than the score of the pilot study (α = 0.413). The internal consistency between the MSPSS-12 and PHQ-9 scores improved from the pilot study to the current study, which indicates that the measures were reliable and valid. In addition, SMAQ-6 score of the larger study was significantly higher (α = 0.646) than the pilot study (α = 0.345), reflecting acceptable reliability coefficient.

### Ethical considerations

Ethical clearance for this study was obtained from the University of KwaZulu-Natal’s Humanities and Social Sciences Research Ethics and Health Research Review Board Ethics Committee of KwaZulu-Natal Department of Health. All human participants granted written consent before enrolling in the study. (HSSREC/00000607/2019); HSSREC-13 December 2019.

## Results

### Demographic and descriptive statistics

Table 1 shows the socio-demographic characteristics of the sample. Out of the 201 participants enrolled in the study, most participants were females (*n* = 142, 70.6%). The participants had a mean age of 39.3 years (SD = 12.1), with the majority never having been married (*n* = 147, 73.1%), while 97 participants (48.3%) had completed secondary education. Additionally, a significant proportion of participants were unemployed (*n* = 132, 65.7%) and most demonstrated high ART adherence (*n* = 108, 53.7%), experienced depression (*n* = 123, 61.2%) and reported high perceived social support (50.7%). The average score of depressive symptoms was 10.2 (SD = 4.6) with just over one-third (*n* = 65, 32.3%) having scores of 5–9 indicative of mild depression and (*n* = 58, 28.9%) having scores greater than 9 suggestive of clinical depression. The mean score of perceived social support was 63.6 (SD = 20.2), with nearly half (*n* = 94, 46.8%) of respondents having scores below the median observed to have low levels of perceived social support, while half (*n* = 102, 50.7%) had high levels of perceived social support with scores at and/or above the median (see Table 1).

The mean score of ART adherence was 6.2 (SD = 1.3), with almost half (*n* = 93, 46.3%) of participants reporting low ART adherence, with scores below the median, while over half (*n* = 108, 53.7%) of participants had high ART adherence with scores at and/or over the median (see Table 1 & Table 2). The mean scores and corresponding SD derived from the sample data suggest moderate levels of depressive symptoms (M = 10.2, SD = 4.6) and ART adherence (M = 6.2, SD = 1.3). Conversely, participants reported high levels of perceived social support (M = 63.6, SD = 20.2) as detailed in Table 2.

**TABLE 2:** Means, standard deviations, and inter-correlations among study variables.

Variables	Mean	Standard deviation	Depressive symptoms	Social support	ART adherence
Depressive symptoms	10.2	4.6	1	−0.037	−0.180*
Social support	63.6	20.2	−0.037	1	0.145*
ART adherence	6.2	1.3	−0.180*	0.145*	1

ART, antiretroviral therapy.

* , *p* < 0.05 (2-tailed).

### Bivariate analysis

Statistically significant differences were observed in only two demographic characteristics between low-adherent and high-adherent participants (see Table 1). Participants significantly differed on ART adherence based on age (χ^2^ [5] = 10.86, *p* = 0.054) and employment status (χ^2^ [3] = 8.75, *p* = 0.033), whereas sex (χ^2^ [1] = 0.53, *p* = 0.468), marital (χ^2^ [4] = 7.20, *p* = 0.126) and education status (χ^2^ [5] = 3.51, *p* = 0.320) did not significantly differ in relation to ART adherence. We also tested differences in depressive symptoms (χ^2^ [4] = 16.14, *p* = 0.003) and social support (χ^2^ [1] = 2.80, *p* = 0.094), but social support did not reach significance, while depressive symptoms were significantly different between low-adherent and high-adherent participants (see Table 1). Bivariate correlations (Spearman’s rank order correlation coefficients) are presented in Table 2. Depressive symptoms were significantly and negatively correlated with adherence to ART (rs = -0.180, *p* = 0.046), while social support was significantly and positively associated with adherence to ART (rs = 0.145, *p* = 0.043). Both social support and depressive symptoms were not significantly related to each other (rs = -0.037, *p* = 0.686).

### Main and interaction effects

Binary logistic regressions of depressive symptoms and social support on ART adherence were performed without and with interaction terms (see Table 3). Social support was not significantly associated with ART adherence in the regression model without the product term (B = 0.007; odds ratios [OR 1.007; 95% Confidence intervals [CI] = 0.989, 1.025; *p* = 0.475). The statistical test indicated a significant inverse association between depressive symptoms and ART adherence (B = -0.105; 0R 0.901; 95%CI = 0.827, 0.981; *p* = 0.016) in the main effect model without the interaction term. The observed OR value of 0.901 between depressive symptoms and ART adherence suggests that higher levels of depression were associated with lower levels of ART adherence by a multiplicative factor of about 0.90 (see Table 3). The product term of depressive symptoms and social support was also added to the regression model to examine the synergistic effect of these predictor variables on ART adherence (see Table 3). The interaction between depressive symptoms and social support on ART adherence was statistically significant (B = -0.006; 0R 0.994; 95%CI = 0.989, 1.000; *p* = 0.037), which suggest that the effect of depressive symptoms on ART adherence depends on the values of social support. An OR value of 0.994 for the interaction term indicates a comparatively weaker association of social support and depressive symptoms with ART adherence. However, this indicates that among PLHIV with depressive symptoms the odds of ART adherence decrease by a factor of 0.99 for every unit increase in social support scores.

**TABLE 3:** Interaction between depression, and social support on antiretroviral therapy adherence in logistic regression.

Variable	Adherence to ART				*p*
	B	SE	OR	95% CI	
**Main effect model**					
Depressive symptoms	−0.105	0.044	0.901	0.827, 0.981	0.016
Social Support	0.007	0.009	1.007	0.989, 1.025	0.475
**Interaction model**					
Depressive symptoms	−0.102	0.046	0.903	0.826, 0.987	0.025
Social support	0.001	0.010	1.001	0.981, 1.020	0.959
Interaction term	−0.006	0.003	0.994	0.989, 1.000	0.037

ART, antiretroviral therapy; B, unstandardised coefficient; SE, standard error; OR, odds ratios; CI, confidence intervals.

## Discussion

This study aimed to explore how social support and depressive symptoms interact to influence ART adherence among adult PLHIV in SA. This research adds to the existing literature on the complex and bidirectional relationship between social support, depression, and ART adherence.

In light of Beck’s cognitive theory of depression, the study hypothesised that depressive symptoms predict ART adherence among adult PLHIV in SA. The results of the study showed that there was a statistically significant association between depressive symptoms and ART adherence. This result confirmed the hypothesis. Additionally, a statistically significant difference was found between depressive symptoms and ART adherence. This result was significant, further supporting the hypothesis and indicating that depressive symptoms can profoundly affect ART adherence. This result was consistent with what has been observed in the Republic of Congo (Ekat et al. 2020), in Uganda (Wagner et al. 2020) and elsewhere (Andini, Yona & Waluyo 2019; Shearer et al. 2018; Mao et al. 2019; Spielman et al. 2021), where depressive symptoms were found to be a significant predictor of poor ART adherence.

This suggests that those with severe depressive symptoms were less likely to adhere to their ART regimen than those with less severe symptoms. However, a study conducted over a decade ago in SA by Kitshoff et al. (2012), found different results. They surveyed 146 adult PLHIV on ART in the KZN province and found no significant association between depressive symptoms and poor ART adherence. Although the study found no significant association between depressive symptoms and poor ART adherence, it is imperative to note that this study has become outdated. The inconsistencies in findings between this study and the previous study can be attributed to variations in sample characteristics, sample size, methodological approaches, and geographical location (Dattani et al. 2022), given their potential influence on the relationship between depressive symptoms and ART adherence.

Beck’s Cognitive Theory of Depression has provided insight into the core issues underlying depressive symptoms and the interpretation of findings in this study. Beck theorised that depressed people focus on the negative aspects of events, interactions and situations, overlooking any evidence of positive outcomes, which further contributes to depressive symptoms (Beck 1967). For instance, this theory states that depressed individuals living with HIV might have distorted thoughts about themselves, their illness and treatment, which could lead to poor ART adherence. His theory further proposes that by changing these cognitive distortions (Beck 1967), PLHIV can better manage their health conditions, reduce depressive symptoms and improve ART adherence.

The study further utilises SCT to underscore the critical role of environmental factors in influencing behaviour, highlighting its significance in shaping the extent of social support that individuals receive. This theory is integral to this study as it further provides insight regarding how social support plays an essential role in the context of external factors influencing behaviour (Bandura 2001). This theory is useful in understanding the mechanisms by which external factors such as social support and internal factors such as depressive symptoms interact to influence individual behaviour such as ART adherence and outcomes (Bandura 2001). Thus, from an eclectic lens of the theory of depression (Beck 1967), SCT (Bandura 1986) and the buffer model (Cohen & Wills 1985), the study postulated that social support could buffer the negative effects of stress and can serve as a protective factor against depressive symptoms and poor ART adherence outcomes.

As a result, the study hypothesised that there is a statistically significant interaction between social support and depressive symptoms in predicting ART adherence. The results from the study showed that the variables tested had a statistically significant interaction in predicting ART adherence. This indicates that the hypothesis was supported by this result. By examining the effect of perceived social support on poor ART adherence, this study further supports the notion of a buffer model (Cohen & Wills 1985), where the presence of social support provides a protective factor either directly or indirectly against poor ART adherence. Contrary to our expectations, this study did not find a significant association between social support and ART adherence with and without the product term in the regression model. This result was consistent with the study conducted in SA by George and McGrath (2018), who found that social support was not significantly associated with ART adherence.

Additionally, this suggests that the buffer model of social support, which states that social support has a protective effect against stress, was not applicable in this case more specifically in terms of the direct effect model (Lakey & Orehek 2011). It may be that the relationship between social support and ART adherence is more complex than the buffer model suggests. Although social support is crucial for treatment adherence (Deshmukh et al. 2018; Shushtari et al. 2022), its effectiveness may be dependent on the quality, quantity and timing of support provided to PLHIV. For example, if the support given is too intrusive or if the support is not given in a timely manner, it can be less effective in helping the patients adhere to their treatment. Our finding of a non-significant association between social support and ART adherence aligns with results of George and McGrath (2018), however a substantial body of evidence has been established demonstrating a significant association between social support and ART adherence (Damulira et al. 2019; Mireles et al. 2023; Oliveira et al. 2020). The divergence in study findings could stem from changes in the accessibility, quality, and perceived significance of social support across different contexts. Factors such as cultural differences, subjective understanding of support, and varying support systems (Zheng et al. 2021) might shape the impact of social support on ART adherence, contributing to divergent results.

Moreover, it was found, however, that the negative interaction effect of depressive symptoms and social support was significantly associated with ART adherence. This result was aligned with findings from just two studies conducted in China (Mao et al. 2019) and Thailand (Mireles et al. 2023). This result indicates that when both high levels of social support and high levels of depression coexist, they jointly influence ART adherence, potentially leading to low adherence levels. In other words, the presence of high levels of depression counteracts the positive effect of high levels of social support in the combined influence on ART adherence, resulting in low ART adherence levels. In this context, despite receiving high levels of social support, individuals with high levels of depression would exhibit low ART adherence levels. Conversely, in the study conducted by Mao et al. (2019), among 319 PLHIV in Guangxi, those with higher levels of social support had a greater reduction in depressive symptoms, which was associated with high levels of ART adherence. More recently, a study by Mireles et al. (2023), highlighted the fact that in a group of 214 young men who had sex with men living with HIV, those with severe depressive symptoms were more likely to benefit from social support, which was associated with improved ART adherence. A key finding of this study suggests that social support might be less effective in facilitating ART adherence among PLHIV, particularly when they experience high levels of depression. In simple terms, for PLHIV who for instance have strong feelings of sadness and hopelessness, getting support from family, friends and or significant others might not be beneficial in helping them adhere to their ART.

The integration of the Social Cognitive Theory, the Buffer Model and the Cognitive Theory of Depression was essential in informing our understanding of why a link exists between social support and ART adherence among individuals with depressive symptoms in the context of our study. Therefore, while social support could be helpful in improving ART adherence for certain individuals with depression, it is essential to explore additional factors that might also have potential to reduce depressive symptoms.

### Limitations and strengths

Firstly, this study targeted only adult PLHIV between the ages of 18 and 75 years, and many participants were females from semi-urban regions of Durban. Furthermore, the study was conducted in a single province, and thus our results may not be generalisable to other parts of SA. Secondly, the self-reported measures may not accurately capture the true condition of the participants as they are not directly observed or tested. This may lead to inaccurate responses that may not accurately reflect the true condition of the participants. Thirdly, it was not clear in this study what type of support the participants had received from each specific source as social support is a multidimensional construct. The type of support perceived from each source can vary greatly from emotional, practical, informational or appraisal (Cohen 2004). Knowing this information could provide valuable insight into the overall effectiveness of social support on individuals’ mental health and well-being. Finally, the study gathered cross-sectional data which did not allow the description of relationship patterns between study variables over time. To further understand the complexity of these relationships, future studies should incorporate longitudinal data.

Despite affirmed limitations, the strength of this study lies in the intricate and complex interplay between social support and depressive symptoms and how they influence ART adherence within the South African context. It also identified potential avenues to improve interventions to support ART adherence among PLHIV in SA.

### Practical implications

This study highlights the significance of a holistic support framework for PLHIV. While acknowledging the pivotal role of healthcare professionals in addressing the psychosocial needs of PLHIV, this study recommends a multi-dimensional approach. Firstly, considering the evidence suggesting the dearth of depression diagnosis within healthcare facilities, the study recommends robust mental health awareness and regular screening practices within healthcare settings. Secondly, recognising that social support extends beyond clinical settings, the study advocates for family and community-based initiatives. These initiatives could encompass a range of strategies such as support groups that facilitate the sharing of experiences and sense of solidarity among PLHIV and community-based educational programs that could provide key information on mental health, ART adherence and overall well-being. Additionally, the study suggests family involvement as a primary component within these efforts and it stands out as a basic pillar in fostering a supportive and enabling environment that contributes to overall well-being and better health outcomes of PLHIV. Such a comprehensive approach will better cater to the multifaceted needs of PLHIV and promote better levels of ART adherence.

## Conclusion

The study has shown that the PLHIV with depressive symptoms were less likely to adhere to their ART than those without symptoms, suggesting that depression had a negative direct effect on ART adherence. The second key finding indicates that when depressive symptoms and social support were both present, the positive effect of social support on ART adherence was weakened because of the negative direction of the interaction effect associated with poor ART adherence outcomes. The evidence from this study suggests that social support did not sufficiently act as a buffer against the negative effect of depression on ART adherence in this population. A further study should delve into more in-depth exploration of specific contextual factors, such as sociodemographic influences including but not limited to age and employment status. Exploring these factors can provide insights into how sociodemographic factors contribute to the complex interplay between depression, social support and ART adherence.
